# Effect of visual disruption on the lower limb kinematic and kinetic characteristics in athletes after anterior cruciate ligament reconstruction

**DOI:** 10.3389/fbioe.2026.1767092

**Published:** 2026-06-08

**Authors:** Peng Yu, Yonghong Yu, Jiahao Sun, Yuxin Chen, Ziyue Zhou, Yuan Tang

**Affiliations:** 1 Wuhan Sports University, Wuhan, Hubei, China; 2 College of Sports Medicine, Wuhan Sports University, Wuhan, Hubei, China; 3 College of Physical Education, Wuhan Sports University, Wuhan, Hubei, China; 4 College of Sports Training, Wuhan Sports University, Wuhan, Hubei, China; 5 Department of Sports Medicine, Wuhan Orthopedic Hospital of Integrated Traditional Chinese and Western Medicine (Affiliated Hospital of Wuhan Sports University), Wuhan, Hubei, China

**Keywords:** anterior cruciate ligament, biomechanics, cutting, sensory reweighting, stroboscopic glasses

## Abstract

**Background:**

Proprioceptive deficits following anterior cruciate ligament (ACL) injury increase reliance on visual input in athletes after ACL reconstruction (ACLR). Visual disruption may therefore alter movement patterns and increase re-injury risk. However, its influence on lower limb biomechanics during cutting maneuvers in ACLR athletes remains unclear.

**Purpose:**

To investigate the effects of visual disruption on the kinematic and kinetic characteristics of the lower limb during the 90° cutting maneuver in athletes after ACLR.

**Methods:**

Twenty athletes after ACLR and twenty healthy athletes were recruited to randomly undergo two different visual conditions, eyes-open and visual disruption, and to complete the 90° cutting maneuver in each of the two visual conditions. Visual disruption was performed with strobe glasses. A nine-camera infrared motion capture system (Vicon T40, 200 Hz) was used to collect lower-limb kinematics data during the 90° cutting task, while a three-dimensional force platform (Kistler, 1,000 Hz) recorded kinetic data. Data were processed using Visual 3D software, and statistical analyses were conducted using SPSS (version 25.0). A two-factor repeated measures analysis of variance was used to determine the effects of group and visual conditions on kinematic and kinetic variables.

**Results:**

(1) Compared with the eyes-open condition, the peak knee valgus angle (P = 0.025, ES = 0.157) and peak ankle inversion angle (P = 0.005, ES = 0.233) of athletes after ACLR were significantly increased under visual disruption conditions. There was no significant difference between the kinematic variables of the healthy athletes in the two visual conditions (*P* > 0.05). (2) Compared with the eyes-open condition, the hip extension moment of athletes after ACLR was significantly increased (*P* = 0.037) and the knee extension moment was significantly reduced (*P* = 0.039) under visual disruption conditions. There was no significant difference (*P* > 0.05) in the kinetic variables of the healthy athletes between the two visual conditions.

**Conclusion:**

Visual disruption increased knee valgus and ankle inversion angles in athletes after ACLR, which may increase the risk of secondary anterior cruciate ligament injuries and lateral ankle sprains. These results suggest that ACLR athletes have a poorer ability to recalibrate sensory information to visual disruption compared to healthy athletes.

## 1 Introduction

Anterior cruciate ligament (ACL) rupture is one of the most common and severe knee injuries in sports. Although ACL injuries may occur through contact mechanisms, most are non-contact injuries arising during high-demand tasks such as cutting, pivoting, and landing (Montalvo et al., 2019). While ACL reconstruction restores mechanical stability, graft reinnervation is often incomplete or delayed, and mechanoreceptor loss may result in persistent proprioceptive deficits (Paschos and Howell, 2016). However, although surgery can restore the macrostructure of the ligament, it is difficult to compensate for the loss of mechanoreceptors caused by ligament injury (Zhang et al., 2016; Lee et al., 2020). Lacking the afferent sensory organs of the original ligament, reinnervation is often incomplete or delayed. As a result, even after successful structural repair, proprioceptive function may remain impaired (Relph et al., 2014; Ageberg and Fridén, 2008). Proprioceptive deficits may trigger a series of alterations that ultimately lead to movement impairment (Dingenen et al., 2015; Tecco et al., 2006) and abnormal biomechanical characteristics (Grooms et al., 2015). Studies have shown that proprioceptive dysfunction is associated with poor movement quality (Cronström and Ageberg, 2014) and an increased risk of knee osteoarthritis in patients with ACLR (Cinque et al., 2018).

Human locomotion is based on sensorimotor control, i.e., the dynamic interplay between sensory afferents in the somatoreceptors, information processing in the central nervous system, and motor behavior (Baumeister et al., 2012). In this model, sensory information provides the possibility to adapt to and interact with the environment. Visual stimuli, together with proprioceptive and vestibular sensory information, play an important role as a source of information and provide sensorimotor feedback during the planning and execution of movement (Santel and lo, 2005; Patla and Vickers, 1997). Damage to any of these three sensory systems requires compensation from the remaining sensory systems (Patla and Vickers, 1997). Thus, in individuals with impaired proprioception following ligament injury, compensation from the visual and vestibular systems is required (Nyland et al., 2011), which is defined as sensory reweighting. In individuals with proprioceptive deficits following ACL injury, visual input plays a critical role in maintaining postural stability and coordinating dynamic movements. Evidence indicates that athletes after ACL reconstruction exhibit increased reliance on visual information during postural control and dynamic tasks, reflecting an adaptive shift in sensory weighting (Wikstrom et al., 2017; Louw et al., 2015). This may be due to the loss of ligament mechanoreceptors impairing proprioception and functional stability (Dhillon et al., 2011). Thus, the central nervous system may employ compensatory stabilization strategies to reweight sensation to more reliable stimuli (e.g., visual and vestibular) (Grooms et al., 2015).

During complex sports activities, when athletes must manage multiple variables (e.g., ball, players, field position, game strategy), full visual attention to the environment is required (Hootman et al., 2007; Chaput et al., 2024; Wohl et al., 2021). Directing attention toward the environmental effects of movement, rather than toward one’s own bodily mechanics, generally enhances motor performance (Wulf et al., 2010). However, this attentional allocation may be especially challenging for athletes after ACLR, as fewer attentional resources are left for neuromuscular function. Once visual feedback is reduced, athletes after ACLR will have a greater reliance on mechanoreceptor and vestibular information. Other remaining sources of sensory information may not provide enough afferent information to fully compensate for the limited visual feedback (Grooms et al., 2015). When sports activities impose limitations, such as increased visual distractions, athletes after ACLR surgery may not be able to manage these limitations, leading to injurious movement patterns and subsequent reinjury. When visual information is disrupted, athletes after ACLR may not effectively adapt to these constraints due to persistent proprioceptive impairments and increased visual reliance. This maladaptation may manifest as high-risk biomechanical patterns (Hewett et al., 2005; Shimokochi and Shultz, 2008).

Accordingly, several experimental paradigms have been used to examine the effects of altered visual input on lower limb biomechanics, including visual occlusion, eyes-closed conditions, and stroboscopic visual perturbation (van Rozendaal et al., 2025). Among these approaches, stroboscopic glasses intermittently disrupt visual input while allowing continued task performance. However, this method does not permit precise quantification of visual load and may result in considerable inter-individual variability in adaptation. Furthermore, the intermittent nature of the perturbation may not fully replicate real-world visual deprivation. Despite these limitations, compared with complete visual occlusion, stroboscopic perturbation maintains task feasibility and ecological validity while selectively challenging visual processing (Hülsdünker et al., 2023). This feature enables participants to perform sport-specific movements under constrained visual conditions without introducing substantial non-visual attentional demands, thereby offering meaningful insight into the role of visual input in regulating knee joint kinematics and kinetics in functionally relevant contexts (Ren et al., 2025). ACL injury is mainly related to abnormal biomechanics (Shahbazi et al., 2025). Specifically, smaller knee flexion angles (Chen et al., 2024a), larger knee extension moments (Dai et al., 2012), larger knee valgus angles and moments (Shimokochi and Shultz, 2008), and larger knee internal/external rotation angles during dynamic tasks have been shown to be risk factors for ACL injury (Miyasaka et al., 2002). Cutting maneuvers are considered to be demanding athletic movements that mimic typical athletic movements and may be related to ACL injury mechanisms (McKay et al., 2001). During cutting, the ACL plays a key role in resisting anterior tibial translation and rotational loads at the knee (Zhang et al., 2024).

Therefore, this study aimed to determine whether visual disruption elicits high-risk lower limb biomechanical patterns during a 90° cutting maneuver in athletes after ACL reconstruction (ACLR) compared with healthy athletes. We hypothesized that visual disruption would compromise the ability of ACLR athletes to adapt to altered sensory input, thereby resulting in high-risk biomechanical characteristics during the cutting task. Specifically, relative to the eyes-open condition, ACLR athletes were expected to exhibit reduced knee flexion angles, increased knee valgus angles, and greater vertical ground reaction forces under visual disruption, whereas healthy athletes were anticipated to demonstrate minimal biomechanical differences between visual conditions.

## 2 Data and methods

### 2.1 Study design

**FIGURE 1:**
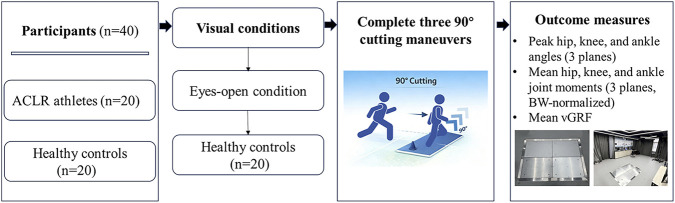
Study flow diagram. ACLR, anterior cruciate ligament reconstruction.

A two-factor factorial repeated-measures design was used in which ACLR athletes and healthy athletes were asked to experience two visual conditions, eyes-open and visual disruption, and to complete three 90° cutting maneuvers in each of the two visual conditions in Figure 1.

### 2.2 Participant

#### 2.2.1 Inclusion criteria

Athletes after ACL reconstruction (ACLR group) were included if they met all of the following criteria (Shahbazi et al., 2025; Khoygani and Esmaeili, 2025):

(1)Unilateral ACL injuries treated with autologous ipsilateral bone patellar tendon-bone or hamstring tendon grafts (semitendinosus and/or gracilis tendon); (2) were cleared to return to all high-level athletic activities by their surgeon and treating rehabilitation specialist and intended to return to cutting and pivoting sports regularly (>50 h/year) (Schmitt et al., 2015); (3) were right-handed, (4) a limb symmetry index of >90% for jump distance in a single-leg horizontal jump test; (5) basketball, soccer, or volleyball player with a pre-injury Tegner score of ≥7.

Inclusion criteria for healthy athletes were (1) no lower limb injuries within 3 months, and (2) basketball, soccer, or volleyball players with a Tegner score ≥7, (3) were right-handed.

#### 2.2.2 Exclusion criteria

Participants were excluded if they had:

(1)Grade III knee ligament injuries, total laminated articular cartilage injuries, history of other arbitrary lower limb surgery, back pain, or lower limb injuries within 3 months; (2) missing data during testing; and (3) inability to complete the assessment as required.

*A priori* power analysis (G*power 3.1.2) was used to determine the appropriate sample size for the whole study. After calculation, we determined the sample size of 38 participants to achieve power = 0.85, effect size = 0.25, and significance level = 0.05 under two-factor repeated measures analysis conditions. Participants were recruited from basketball, soccer, and volleyball, as these sports share comparable non-contact ACL injury mechanisms, including cutting, pivoting, rapid deceleration, and landing tasks. Therefore, 20 athletes after ACLR and 20 healthy athletes were selected for this study. The recruitment period started in 30 September 2023 and ended in 30 January 2024. The basic information of the participants is shown in Table 1.

**TABLE 1:** Basic information of participants.

​	Athletes after ACLR (n = 20)	Healthy athletes (n = 20)	P-value
Sex (female/male, n)	8/12	10/10	0.751
Age (years)	22.0 ± 2.0	21.5 ± 1.8	0.415
Height (cm)	173.3 ± 8.9	176.0 ± 6.6	0.275
Weight (kg)	67.9 ± 9.7	67.3 ± 7.9	0.831
BMI (kg/m^2^)	22.6 ± 2.2	21.7 ± 1.5	0.137
Injured side (left/right, n)	11/9	​	​
Time since surgery (months)	12.5 ± 2.3	​	​
Isolated ACL injury/Concomitant meniscal injury (n)	7/13	​	​

All participants had normal or corrected-to-normal vision and were allowed to wear habitual contact lenses. Individuals with visual impairments were excluded, and none had prior experience with stroboscopic glasses. Familiarization trials were conducted before data collection. All participants were informed about the testing process and signed a written informed consent form. The study was agreed and approved by the medical ethics committee of Wuhan Sports University (No. 2023066).

### 2.3 Experimental data collection

Participants were instructed by the experimenter to familiarize themselves with the test maneuvers and perform a 10-min standardized warm-up before testing. A total of 38 retroreflective markers (14 mm diameter) were placed on the lower limbs based on a modified Plug-in-Gait model. Markers were placed on left and right anterior superior iliac spine, iliac crest, posterior superior iliac spine, greater trochanter of the femur, lateral femoral condyle, medial femoral condyle, lateral malleolus, medial malleolus, heel, first metatarsal head, fifth metatarsal head, and 4 markers on the side of each thigh and shank. All markers were placed by the same experienced investigator to minimize inter-operator variability. Prior to data collection, the motion analysis system was calibrated, and a standing calibration trial was obtained to determine joint centers and to create a segment coordinate system. Visual disruption was induced using stroboscopic glasses (Senaptec Strobe, USA), which intermittently occlude vision by alternating between transparent and opaque states. The strobe glasses were set to 3 Hz (0.1 s on/0.233 s off), which was selected to induce a moderate level of visual disruption that challenges visuomotor processing and neuromuscular control while maintaining the feasibility and safety of the single-leg jumping task (Seunguk et al., 2021; Bodkin et al., 2022). Participants in each group performed three cutting trials with strobe glasses (i.e., the visual disruption condition) and without strobe glasses (i.e., the eyes-open condition) for a total of six trials. The order of the visual conditions (with or without strobe glasses) was randomized. To minimize the influence of limb dominance on the results, athletes who had under-gone ACLR performed the cutting task using their operated limb, whereas healthy athletes were instructed to complete the task using a randomly selected limb. The cutting maneuver consisted of the following two parts: (1) the participant stood at the starting point 3 m behind the force plate and ran down the track toward the force plate as fast as possible, and (2) The participant’s testing limb landed on one side of the force plate and then quickly cut to the contralateral side at a 90° angle in Figure 1. After completing 3 successful cutting maneuvers, the average of the 3 results was selected for analysis (Howard et al., 2025). Each cutting maneuver was separated by 3 min to avoid the influence of fatigue on the experimental results. A nine-camera infrared high-speed motion capture system (Vicon, T40, Vicon, UK, 200 Hz) was applied to record the kinematic parameters of the affected/dominant lower limb during the 90° cutting maneuver, and a three-dimensional force platform (Kistler 9287CA, Kistler Instrumente AG, Winterthur, Switzerland; 1,000 Hz) was applied to record the kinematic parameters.

### 2.4 Experimental data processing

Kinematic and inverse dynamics analysis was performed using Visual 3D (C-Motion, Inc) software. Both marker trajectories and ground reaction forces were filtered using a fourth-order zero-lag Butterworth low-pass filter with a cutoff frequency of 12 Hz prior to inverse dynamics calculations. 3D lower limb models were created using previously described methods (Ford et al., 2010). The model was used to calculate 3D ankle, knee, and hip joint angles and moments. To avoid the possibility that differences in static frontal-plane alignment may account for the observed dynamic differences, we additionally calculated knee frontal-plane angles to examine potential baseline alignment differences between groups, as static alignment has been shown to influence dynamic biomechanical patterns (Zhao et al., 2025). To determine the knee frontal-plane angle during static standing, the anteroposterior components of the ankle, knee, and hip joint centers in the global coordinate system were set to zero to align all joints within the same plane. The mechanical axis angle was then calculated as the arccosine of the two vectors extending from the hip to the knee and from the knee to the ankle joint center (Stief et al., 2020; Kornaropoulos et al., 2010; Mündermann et al., 2008).

Lower extremity joint kinematics were calculated through use of a Cardan rotation sequence in the order of flexion-extension, abduction-adduction, and internal-external rotation. Standard inverse dynamics analysis was used to calculate kinetic variables at the ankle, knee, and hip. Marker trajectories and force plate data were filtered using a low-pass Butterworth digital filter (12 Hz), which is thought to minimize artifacts during inverse dynamics analysis of high-impact activities (Kristianslund et al., 2012; Pozzi et al., 2017). The landing phase was defined as the period from the initial contact with the force plate to the peak knee flexion angle (Lee et al., 2024). Initial contact with the force plate was defined as the 1st moment when the vertical ground reaction force (vGRF) was >50 N (Lepley et al., 2015). Since the landing phase is considered to be the most injury-prone phase of the ACL (Lepley et al., 2015). Therefore, kinematic and dynamic variables were extracted only during the landing phase. The kinematic variables of interest included peak hip, knee, and ankle angles in three planes, and the kinetic variables included average moments of the hip, knee, and ankle joints in three planes and the mean vGRF. The joint angles were determined by the angle between the distal and proximal segments. Inverse kinetics was used to calculate joint moments. All kinetic variables were normalized using body weight. A negative sign (−) indicates joint extension/plantar flexion, eversion/abduction, or external rotation.

### 2.5 Statistical analysis

Statistical processing was performed using SPSS 25.0 statistical software, and P < 0.05 was regarded as a statistically significant difference. Continuous variables were expressed as mean ± standard deviation (SD). Normality was confirmed using the Shapiro-Wilk test, and homogeneity of variance was confirmed using the Levene test. For demographic data and knee frontal-plane angles during static standing, Fisher’s exact probability method was used for comparisons between groups for count data, and independent sample T-test was used for comparisons between groups for measurement data. For kinematic and kinetic data, a two-way repeated measures ANOVA was used to determine the interaction effect of group and visual condition. If an interaction effect was found to be significant, a simple effects analysis was performed and multiple comparisons were made using Bonferroni.

## 3 Results

A total of 40 participants were included in the final analysis, consisting of 20 athletes after ACL reconstruction (ACLR group) and 20 healthy athletes (healthy group). There were no significant differences between groups in sex distribution, age, height, weight, or body mass index (all P > 0.05). In the ACLR group, the mean time since surgery was 12.5 ± 2.3 months, with 11 participants having left-side and 9 having right-side reconstruction. Detailed demographic characteristics are presented in Table 1.

### 3.1 Kinematic variables

Independent-samples t-test results showed no significant difference in knee frontal-plane angle during static standing between ACLR athletes (−0.59° ± 2.63°) and healthy athletes (−0.78° ± 2.06°) (*P* = 0.821, t = 0.229).

The two-way ANOVA revealed significant group × visual condition interaction effects for peak knee valgus angle (*P* = 0.025, *F* = 5.594, ES = 0.157) and peak ankle inversion angle (*P* = 0.005, *F* = 9.090, ES = 0.233). Post hoc analysis showed that compared with the eyes-open condition, the peak knee valgus angle (*P* < 0.001) and peak ankle inversion angle (*P* < 0.001) of athletes after ACLR were significantly increased under visual disruption conditions. There was no significant difference in the peak knee valgus angle (*P* = 0.161) and peak ankle inversion angle (*P* = 0.431) of healthy athletes under the two visual conditions (Table 2).

**TABLE 2:** Kinematic variables.

​	ACLR athletes		Healthy athletes		Group × condition *P*, *F* and ES	Groups *P*, *F* and ES	Condition *P*, *F* and ES
	Visual disruption	Eyes-open	Visual disruption	Eyes-open			
Peak hip angle (°)							
Flexion/extension	30.47 ± 5.20	29.50 ± 5.82	24.02 ± 6.61	23.80 ± 6.90	0.431, 0.637, 0.021	0.008*, 8.132, 0.213	0.209, 1.646, 0.052
Adduction/abduction	2.44 ± 5.35	3.10 ± 6.64	1.83 ± 5.44	2.41 ± 4.81	0.935, 0.007, <0.001	0.737, 0.115, 0.004	0.205, 1.669, 0.053
Internal/external rotation	−6.44 ± 8.08	−6.25 ± 8.25	−5.52 ± 6.75	−5.63 ± 6.87	0.816, 0.055, 0.002	0.768 0.088, 0.003	0.949, 0.004, <0.001
Peak knee angle (°)							
Flexion/extension	27.69 ± 4.77	27.11 ± 4.68	32.20 ± 6.40	30.90 ± 6.61	0.481, 0.072, 0.002	0.041*, 1.855, 0.058	0.075, 0.749, 0.024
Varus/valgus	−4.51 ± 2.39	−3.19 ± 1.66	−3.58 ± 2.04	−3.19 ± 1.83	**0.025** ^#^, 5.594, 0.157	0.498, 0.470, 0.015	<**0.001**, 19.332, 0.392
Internal/external rotation	5.82 ± 5.57	5.42 ± 3.93	5.13 ± 4.97	4.22 ± 4.07	0.666, 0.191, 0.006	0.547, 0.369, 0.012	0.277, 1.224, 0.039
Peak ankle angle (°)							
Dorsiflexion/plantarflexion	6.33 ± 4.51	5.77 ± 4.06	5.78 ± 5.70	5.56 ± 5.06	0.814, 0.056, 0.002	0.811, 0.058, 0.002	0.596, 0.285, 0.009
Varus/valgus	15.30 ± 4.34	13.62 ± 3.70	14.21 ± 4.48	13.95 ± 3.72	**0.005** ^#^, 9.090, 0.233	0.791, 0.071, 0.002	<**0.001**, 17.164, 0.364
Internal/external rotation	3.17 ± 3.50	2.02 ± 4.69	2.84 ± 2.64	2.53 ± 2.39	0.561, 0.343, 0.011	0.925, 0.009, <0.001	0.316, 1.040, 0.034

Values are expressed as mean ± SD., Significance level was set at P < 0.05.

*Indicates a significant main effect of group. # indicates a significant interaction effect (Group × Condition).

Positive values indicate flexion, abduction, and internal rotation, whereas negative values indicate extension, adduction, and external rotation.

Cells in bold print indicate significant results.

No significant group × visual condition interaction effects were observed for peak hip flexion angle (*P* = 0.431, *F* = 8.132, ES = 0.213) or peak knee flexion angle (*P* = 0.481, *F* = 1.855, ES = 0.058). However, there was a significant main effect of group (*P* = 0.008, *P* = 0.041). Athletes with ACLR had higher peak hip flexion angle (*P* < 0.001) and lower peak knee flexion angle (*P* = 0.001) compared with healthy athletes (Table 2).

### 3.2 Kinetic variables

The two-way ANOVA revealed no significant group × visual condition interaction effects for hip extension torque (*P* = 0.901, *F* = 0.016, ES = 0.001) or knee extension torque (*P* = 0.643, *F* = 0.219, ES = 0.007). However, there were significant main effects of condition and group (*P* < 0.05). Compared with the eyes-open condition, visual distraction significantly increased hip extension moment (*P* = 0.006) and decreased knee extension moment (*P* = 0.015). Compared with healthy athletes, athletes after ACLR had higher hip extension torque (*P* = 0.035) and lower knee extension torque (*P* = 0.034) (Table 3).

**TABLE 3:** Kinetic variables.

​	ACLR athletes		Healthy athletes		Group × condition *P*, *F* and ES	Groups *P*, *F* and ES	Condition *P*, *F* and ES
	Visual disruption	Eyes-open	Visual disruption	Eyes-open			
Hip moment (Nm/kg)							
Flexion/extension	−1.00 ± 0.25	−0.92 ± 0.25	−0.84 ± 0.17	−0.76 ± 0.17	0.901, 0.016, 0.001	0.035*, 4.871, 0.140	**0.006** ^**#**^, 8.811, 0.027
Adduction/abduction	0.67 ± 0.40	0.62 ± 0.33	0.57 ± 0.42	0.54 ± 0.35	0.530, 0.092, 0.003	0.698, 0.403, 0.013	0.236, 1.475, 0.047
Internal/external rotation	−0.35 ± 0.94	−0.33 ± 0.36	−0.31 ± 0.52	−0.27 ± 0.48	0.930, 0.007, <0.001	0.774, 0.084, 0.003	0.806, 0.062, 0.002
Knee moment (Nm/kg)							
Flexion/extension	−0.98 ± 0.28	−1.06 ± 0.27	−1.21 ± 0.30	−1.27 ± 0.30	0.643, 0.219, 0.007	0.034*, 4.949, 0.142	**0.015** ^**#**^, 6.676, 0.182
Varus/valgus	−0.58 ± 0.31	−0.54 ± 0.23	−0.55 ± 0.30	−0.51 ± 0.25	0.992, 0.001, <0.001	0.769, 0.087, 0.003	0.282, 1.335, 0.043
Internal/external rotation	0.11 ± 0.12	0.11 ± 0.12	0.10 ± 0.07	0.10 ± 0.07	0.720, 0.044, 0.001	0.886, 0.123, 0.004	0.769, 0.074, 0.002
Ankle moment (Nm/kg)							
Dorsiflexion/plantarflexion	−0.87 ± 0.27	−0.86 ± 0.23	−0.82 ± 0.31	−0.81 ± 0.18	0.973, 0.001, <0.001	0.415, 0.682, 0.022	0.943, 0.005, <0.001
Varus/valgus	0.73 ± 0.23	0.71 ± 0.22	0.71 ± 0.30	0.68 ± 0.30	0.724, 0.085, 0.003	0.798, 0.067, 0.002	0.082, 3.121, 0.094
Internal/external rotation	0.19 ± 0.09	0.18 ± 0.09	0.17 ± 0.08	0.17 ± 0.10	0.819, 0.075, 0.002	0.524, 0.417, 0.014	0.542, 0.443, 0.015
vGRF (BW)	1.76 ± 0.20	1.76 ± 0.29	1.73 ± 0.18	1.71 ± 0.22	0.846, 0.039, 0.001	0.576, 0.316, 0.010	0.788, 0.073, 0.002

Values are expressed as mean ± SD, and normalized to body weight. Significance level was set at P < 0.05.

*Indicates a significant main effect of group. and indicates a significant main effect of visual condition. vGRF, indicates vertical ground reaction force; BW, indicates body weight.

Positive values indicate flexion, abduction, and internal rotation, whereas negative values indicate extension, adduction, and external rotation.

Cells in bold print indicate significant results.

## 4 Discussion

When vision is disrupted, athletes after ACLR may be unable to cope with this limitations due to proprioception deficits and visual reliance, leading to impaired movement patterns and re-injury. However, it is unclear whether visual interference alters lower extremity biomechanics during cutting movements in athletes after ACLR. The purpose of this study was to investigate the effects of visual disruption on the lower limb biomechanical characteristics of the cutting maneuver in athletes after ACLR and healthy athletes. The primary findings were that visual disruption led to significant increases in knee valgus angle (ES = 0.157) and ankle inversion angle (ES = 0.233) exclusively in athletes after ACLR, whereas healthy athletes demonstrated stable kinematic patterns across visual conditions. In addition, visual disruption significantly increased hip moment and decreased knee extension moment in both ACLR and healthy athletes. In other words, visual disruption only affected lower limb kinematics during cutting in athletes after ACLR, suggesting that athletes after ACLR are less able to reweight sensory information when visually disturbed.

### 4.1 Kinematics

As we hypothesized, only athletes after ACLR exhibited altered movement patterns when visually disturbed during cutting. Specifically, visual disruption increased knee and ankle valgus angles during the landing phase in ACLR athletes. Increased knee valgus angle has been identified as one of the most important risk factors for ACL injury (Chen et al., 2024b; Hewett et al., 2006). This result suggests that visual disruption causes ACLR athletes to exhibit a more injurious movement pattern in the coronal plane. Altered movement patterns in patients with ACLR due to reduced visual information have been frequently reported in the literature (Lee et al., 2024; Han et al., 2022). Miko et al. (Miko et al., 2021) noted that postural stability in ACLR patients was significantly impaired under eyes-closed conditions compared to eyes-open conditions. This may indicate that unique neural processing deficits persist after ACLR. Another study pointed that lack of vision resulted in reduced absolute velocity and displacement, especially for participants with ACLR (Bjornaraa and Di Fabio, 2011). Although these previous studies involved different populations and/or tasks, our results are similar to their observations. Our study suggests that ACLR athletes may not be able to rebalance other sensory inputs to compensate for reduced visual information. The impaired somatosensory system of ACLR athletes may inhibit effective and safe movement patterns when visual information is limited.

In contrast, healthy athletes appear to have the ability to reweigh other sensory information. Song et al. (Song et al., 2017) found that the reduction in plantar skin sensation caused by ice immersion did not affect postural control in healthy individuals. This result is similar to the findings of a previous study, which found that plantar sensory reduction did not negatively affect postural control in healthy young adults while standing on one leg, regardless of vision status (McKeon and Hertel, 2007). Similarly, Corbin et al. (McKeon and Hertel, 2008) found that young healthy adults had no postural control deficits in single-legged balancing with eyes closed after using textured insoles. Overall, these results suggest that uninjured, healthy athletes appear to be able to successfully reweight somatosensory and/or vestibular inputs to compensate for the loss of visual information and plantar somatosensory inputs.

The results of this study also showed that visual disruption increased ankle inversion angle in athletes after ACLR. Increased inversion angle during landing has been identified as a risk factor for ankle sprains (Grooms et al., 2018). This suggests that visual disruption increases the risk of ankle sprain in athletes after ACLR to some extent. This can be explained from the following perspectives. First, the lower limbs act as a kinetic chain, any alteration in the kinematics of the knee would likely affect the hip and ankle (X et al., 2022). An increase in knee valgus angle may increase the offset of the center of gravity in the coronal plane and place the ankle in a more varus position (Emamvirdi et al., 2023). A recent study also confirmed a correlation between ankle lateral ligament sprains and ACL injuries (X et al., 2022). Second, visual feedback may provide greater input to maintain neuromuscular control after ligament injury. Reduced visual feedback may cause athletes after ACLR to use a cautious landing strategy, contacting the ground in a more plantarflexed and inverted ankle position as a means of delaying the arrival of peak ground reaction forces (Louw et al., 2015). Additionally, reduced visual feedback may lead to impaired neuromuscular control of the knee and/or ankle in ligament-injured individuals, making it difficult to control coronal plane excursions (Lee et al., 2024; Grooms et al., 2018).

### 4.2 Kinetics

The results of this study showed that both ACLR athletes and healthy athletes showed higher hip extension moment and lower knee extension moment under visual disruption conditions compared with eyes-open conditions. This can be explained by the fact that out of fear of ACL injury/re-injury, athletes tend to transfer knee loads to the ipsilateral hip during landing tasks (Wren et al., 2018). Chen et al. (Chen et al., 2024a) showed that ACLR athletes exhibited lower knee extension moments, higher hip extension moments, and ankle plantarflexion moments during a single-leg landing task compared to healthy athletes. Paradoxically, this avoidance strategy may inhibit normal cartilage-producing stimuli and increase the long-term risk of osteoarthritis in the operated knee joint (Oiestad et al., 2010).

It is worth mentioning that there were differences in hip extension moment and knee extension moment between the two groups. Visual disruption increased hip extension moment while reducing knee extension moment in both groups, indicating a proximal shift in load absorption strategy. In contrast, there was no significant difference in ankle plantar flexion moment between ACLR athletes and healthy athletes under both visual conditions. Which is not exactly consistent with the compensatory pattern of athletes after ACLR in previous studies (Chen et al., 2024a). Differences in motor tasks may provide a plausible explanation for this inconsistency. Compared with single-leg hopping and Bilateral vertical jumping, lateral cutting increases the risk of frontal plane injury. Due to the greater strength of the muscles around the hip joint, the loads that can be cushioned and absorbed are also greater. In order to circumvent injury risk, so post ACLR athletes tend to transfer loads to the more reliable hip joint rather than the ankle joint (Sritharan et al., 2022). Boo et al. (Sritharan et al., 2022) have shown that ACLR athletes exhibit higher hip energy absorption and lower ankle energy absorption during the landing phase compared to healthy athletes. A study on ankle instability showed similar proximal adaptations, Kim et al. (Kim et al., 2018) reported that during the landing maneuver, individuals with chronic ankle instability had lower knee and ankle moments and higher hip extension moments than healthy controls, possibly to compensate for the unreliable sensorimotor system in the ankle.

### 4.3 Clinical implications

In the present study, visual perturbation altered movement patterns in athletes following ACL reconstruction, leading to biomechanical characteristics associated with increased injury risk. Specifically, greater knee valgus and modified hip–knee load distribution under visual constraint may reduce movement efficiency and compromise joint stability during functional tasks. In real-world sport settings, visual input is often limited or divided due to multitasking demands, environmental complexity, or fatigue (Laurent et al., 2025; Li et al., 2026). This suggests that ACLR athletes are less able to recalibrate sensory information to accommodate visual distraction. These findings support the idea that ACLR rehabilitation should not only focus on strength deficits but also address visual dependence. The present findings underscore the potential detrimental effects of excessive visual dependence in athletes following ACL reconstruction. Specifically, increased knee valgus and ankle inversion angles observed under visual disruption may elevate the risk of secondary ACL injury and lateral ankle sprain. Accordingly, incorporating stroboscopic glasses into training programs may help reduce visual reliance and promote more effective sensory reweighting, thereby mitigating these maladaptive movement patterns. Wearing stroboscopic glasses during training can help induce adaptive neuroplastic responses (Grooms et al., 2015; Wohl et al., 2021). When participants train under conditions where their vision is obstructed, the central nervous system undergoes sensory reweighting to maintain their balance, thereby increasing the weight of proprioception (Grooms et al., 2015). Stroboscopic glasses have been used in training programs for various sports, including baseball (Clark et al., 2012), ice hockey (Mitroff et al., 2013), and badminton (Hülsdünker et al., 2021a; Hülsdünker et al., 2021b). Previous studies have shown that upregulating the input to the somatosensory system and reducing visual input through stroboscopic visual training is crucial for improving athletes’ sports performance and reducing the risk of injury (Hülsdünker et al., 2021a; Hülsdünker et al., 2021b).

### 4.4 Limitations

There are several limitations to this study. First, we did not analyze males and females separately due to the relatively small sample size, although some evidence of biomechanical differences between the sexes existed (Hewett et al., 2006). Second, because the participants recruited for this study were young and active individuals, the current findings can only be generalized to the physically active and young population. Third, due to the limitations of cross-sectional studies, it is not clear whether ACLR athletes were already sensitive to visual disruption in dynamic movements before the ACL injury, or whether these changes were caused by the ACL injury. Fourth, our exclusion criteria did not include a history of concussion or damage to the vestibular system, which may affect vestibular involvement and/or proprioceptor feedback. Fifth, this study did not assess lower limb biomechanical characteristics during tasks such as level walking. A more comprehensive evaluation across different tasks may help to better understand movement adaptation mechanisms under varying task demands. Finally, due to the relatively small sample size, males and females were not analyzed separately, although there was some evidence of biomechanical differences between the sexes (Hewett et al., 2006).

## 5 Conclusion

An important insight from this study is that ACLR demonstrate a reduced capacity to recalibrate sensory feedback, leading to altered lower limb movement strategies, which may increase the risk of secondary ACL injuries and lateral ankle sprains. Specifically, visual disruption increased knee valgus and ankle valgus angles in athletes after ACLR, Importantly, these biomechanical alterations may not only increase the risk of secondary anterior cruciate ligament injury but also heighten susceptibility to injuries at adjacent joints, particularly lateral ankle sprains, during advanced sport-specific tasks that involve rapid directional changes and limited visual availability. Collectively, these findings indicate that athletes after ACL reconstruction exhibit a diminished capacity for sensory reweighting when exposed to visual perturbation compared with healthy athletes. These results suggest that ACLR athletes have a poorer ability to recalibrate sensory information to visual disruption compared to healthy athletes.

## Data Availability

The original contributions presented in the study are included in the article/supplementary material, further inquiries can be directed to the corresponding author.
